# Cyclin D1, cyclin E, and p21 have no apparent prognostic value in anal carcinomas treated by radiotherapy with or without chemotherapy

**DOI:** 10.1038/sj.bjc.6602111

**Published:** 2004-08-03

**Authors:** A S Allal, P Gervaz, M-A Bründler

**Affiliations:** 1 Division of Radiation Oncology, University Hospital of Geneva, 24 rue Micheli-du-Crest, 1211 Geneva 14, Switzerland; 2 Department of Surgery, University Hospital of Geneva, 1211 Geneva Switzerland; 3 Division of Clinical Pathology, University Hospital of Geneva, 1211 Geneva, Switzerland

**Keywords:** radiotherapy, chemotherapy, predictive, P21, cyclin D, cyclin E

## Abstract

The purpose of this study was to assess the potential prognostic and/or predictive value of the expression of cyclin D1, cyclin E, and p21 protein in a series of 98 anal carcinomas (T1–4, N0–3) treated by radiotherapy with (51) or without (47) chemotherapy in one institution. Correlation with Mib1 index and p53 expression was also investigated. Median follow-up for surviving patients was 124 months (range: 30–266). Immunohistochemical staining was performed on pretreatment biopsies, applying a standard ABC technique for cyclin D1 (clone DSC6, DAKO, 1 : 300), cyclin E (clone 13A3, Novocastra, 1 : 100), p21^WAF/CIP1^ (clone SX118, DAKO, 1 : 50), p53 (clone DO7, DAKO, 1 : 200), and Mib1 (Ki-67, Dianova, 1 : 20). Tumours were classified into low- or high-expression groups according to the expression level of the protein considered. High expression was found in 51% of tumours for cyclin E, in 33.7% for cyclin D1, and in 65% for p21. None of those factors were significantly associated with clinical variables such as advanced T or N categories. In a monovariate analysis, advanced T and N categories and longer overall treatment time were the only variables that correlated significantly with low rate of local control (LC) and disease-free survival. However, in a subgroup analysis, high p21 expression correlated with a trend for significantly higher 5-year LC (87 *vs* 68%, *P*=0.07) in the N0 patients. The results of this study suggest that the cell-cycle proteins investigated are unlikely to be clinically useful in predicting treatment response or prognosis in patients with anal carcinomas.

In anal carcinomas, indicators of patient outcome have traditionally been derived from clinical features, including tumour size/stage, extent of lymph node involvement, and anatomical subsite (Cummings *et al*, 1991; Touboul *et al*, 1994; Allal *et al*, 1997). However, despite careful evaluation of these factors, it is not possible to reliably predict the outcome in individual patients. Anal carcinomas are essentially a loco-regional disease, in which the success of treatment depends mainly upon obtaining local and regional control. While sphincter-conserving approaches yield an appreciable cure rate, loco-regional failure may occur in up to 30–35% of patients (Cummings *et al*, 1991; UKCCCR Anal Cancer Trial, 1996). Thus, identification of new prognostic factors for local–regional control, particularly biological parameters, may allow development of individualised strategies that lead to improved results.

Recently, there has been great interest in tumour proliferation and its relation to disease outcome (Begg *et al*, 1990; Lochrin *et al*, 1992). Besides markers of tumour cell kinetics (tritiated thymidine labelling, S-phase fraction, various proliferation-associated antigens, etc.), studies are now focusing on key elements that regulate cell-cycle progression. Different checkpoints such as the restriction point R in early G1, G1/S, and G2/M transitions are regulated positively by a family of cyclin-dependent kinases (CDKs) and their regulatory subunits, the cyclins (Bartek and Lukas, 2001). Cyclins D and E are both involved in the sequential activation of various CDKs (CDK4/6 for cyclin D and CDK2 for cyclin E), which, if the specific CDK inhibitors are detached from the cyclin–CDK complexes, allows phosphorylation of key substrates such as the retinoblastoma protein and release of transcription factors like E2F. This control system governs the passage of cells from G1 to S phase and initiation of DNA replication (Bartkova *et al*, 1997; Kato, 1999). On the other hand, the CDK inhibitors block cell-cycle transitions by inhibition of the kinase activities of cyclin and CDK complexes. Two families of CDK inhibitors have been identified, the p21^WAF1/CIP1^ family (p21^WAF1/CIP1^, p27^KIP1^, p57^KIP2^) that inhibit a broad range of CDK–cyclin complexes (Elledge and Harper, 1994), and the p16^INK4A^ family (p16^INK4A^, p15^INK4B^, p18^INK4C^, p19^INK4D^) that inhibit CDK4/6 complexes (Sherr and Roberts, 1995). p21 was the first mammalian CDK inhibitor identified. In response to DNA damage, p21 is mainly induced by wild-type p53 and is considered a mediator of the tumour suppressor activity of p53 (el-Deiry *et al*, 1993).

The relation between cyclin/CDK inhibitor expression and prognosis is still a matter of controversy. Cyclin E overexpression was reported to be an indicator of poor outcome in several tumours, including lung cancer (Muller-Tidow *et al*, 2001), non-Hodgkin's lymphoma (Ferreri *et al*, 2001), and breast cancer (Keyomarsi *et al*, 2002). Data on the prognostic value of cyclin D are rather rare, although overexpression was reported to be an adverse factor in hypopharyngeal carcinomas (Nishimura *et al*, 1998). On the other hand, several studies have demonstrated that underexpression of p21 protein is a negative prognostic marker in different malignancies, including lung (Komiya *et al*, 1997), breast (Wakasugi *et al*, 1997), bladder (Stein *et al*, 1998), and ovarian cancers (Anttila *et al*, 1999). In anal carcinomas only one study has been carried out, suggesting a negative impact of reduced expression of p21 (Holm *et al*, 2001). To our knowledge, there has been no previous study assessing the expression of cyclins D and E and their relation with clinical–pathological parameters and patient outcome in anal carcinomas. We studied the potential prognostic and/or predictive value of the expression of cyclin D1, cyclin E, and p21 in a large series of anal carcinomas treated by radiotherapy (RT) with or without chemotherapy in one institution. Expression of these parameters was also correlated with the proliferative index Mib1 and p53 expression.

## PATIENTS AND METHODS

### Patients and samples

The selection of patients for this study was based only on the availability of adequate pretreatment paraffin blocks. Of 196 patients treated from January 1976 to November 1998, we were able to collect 100 adequate embedded biopsy specimens. After exclusion of two patients treated with brachytherapy alone, the study group consisted of 98 patients. All pretreatment biopsies were reviewed by an experienced pathologist (M-A B). All tumours were classified according to the 1997 staging system of the Union Internationale Contre le Cancer (UICC, 1997). Pretreatment characteristics are shown in Table 1

**Table 1:** Patient characteristics (98)

	**No. of patients (%)**
	
Median age, years (range)	68 (41–86)
Male/female (ratio)	22/76 (0.29)
	
*Tumour location*	
Canal ± anorectal junction	72 (73)
Margin	8 (8)
Canal+margin	18 (19)
	
*Histologic type*	
Basaloid and transitional (small cells)	64 (65)
Keratinising squamous (large cells)	31 (32)
Mixed (large and small cells)	3 (3)
	
*TN clinical classification (UICC 1997)*	
T1–2	60 (61)
T3–4	38 (39)
N0	74 (75)
N1–3	22 (23)
Nx	2 (2)

UICC=Union International Contre le Cancer.

.

### Treatment and follow-up

In all patients, treatment was delivered with curative intent at the University Hospital of Geneva, according to schedules whose details have previously been published (Allal *et al*, 1997). RT was delivered in a split course, the first sequence consisting of wide-field external-beam RT (EBRT, 30–40 Gy) and the second of a small-volume boost (EBRT or brachytherapy, 20–22 Gy). The median overall treatment time (OTT) was 72 days (range: 40–261).

Concomitant chemotherapy was administered to 51 patients (52%). Generally chemotherapy started on day 1 and consisted of one cycle of mitomycin C (10 mg m^−2^ intravenous bolus) and a 5-day continuous infusion of 5-fluorouracil (600–800 mg m^−2^ day^−1^). A total of 18 patients received a second course of the same chemotherapy during the boost treatment.

Follow-up information was available for all patients except one, who was lost for follow-up at 30 months without evidence of disease. Information was collected from the medical records for recent patients, while for earlier patients information was provided by their private physician or by contacting the patients themselves. The median follow-up for surviving patients was 124 months (range: 30–266).

### Immunohistochemistry

Immunohistochemical studies were performed on pretreatment biopsies, after review of all slides, to confirm the diagnosis of a squamous cell carcinoma (either of large cell/keratinising, basaloid or mixed type). Formalin-fixed and paraffin-embedded tissue blocks were cut at 4 *μ*m and mounted on silane-coated glass slides. Immunohistochemical stains were performed applying a standard avidin–biotin complex (ABC) technique using the following antibodies: cyclin D1 (clone DSC6, DAKO, 1 : 300), cyclin E (clone 13A3, Novocastra, 1 : 100), p21^WAF/CIP1^ (clone SX118, DAKO, 1 : 50), p53 (clone DO7, DAKO, 1 : 200), and Mib1 (Ki-67, Dianova, 1 : 20). Heat-induced antigen retrieval was performed using a microwave oven (600 W, 3 × 5 min) and citrate buffer (0.01 M, pH6) for all antibodies, except for p53, which was retrieved by using a pressure cooker (3 min). After cooling down and washing, slides were incubated with the primary antibody for 30 min at room temperature. Following another washing cycle, slides were incubated with biotinylated secondary antibody (biotinylated rabbit anti-mouse immunoglobulins, F(ab′)2, DAKO E0413) for 30 min, and after washing with a streptavidin–biotinylated-HRP complex (StreptABComplexes, DAKO K0377). Slides were washed thoroughly, and then incubated with diaminobenzidine used for visualisation.

Evaluation of immunohistochemical stains was carried out by one of the authors (M-A B) blinded to patient outcome. Results were assessed using a semiquantitative score based on the estimated percentage of positive cells: 0=0%; 1=<5%; 2=5–50%; 3=50–90%; and 4=>90%. Tumours were then classified into two groups according to the level of expression of the protein considered (low/negative=low or high/positive=high). Expression of p21 and p53 proteins was considered high if greater than 5% of the malignant cells were stained (score 2–4). For cyclin D1 and cyclin E, any tumour containing stained cells was considered as high expression (scores 1–4), while for Mib-1 only tumours with scores 3–4 were considered as high expression.

### Statistical evaluation

The main end points for this study were local control (LC) and disease-free survival (DFS). Tumour persistence or recurrence in the anorectal area or the perineal skin was considered as events in determining LC, whereas DFS additionally took into account regional nodal recurrences as well as distant metastases. Actuarial LC, overall and DFS rates were calculated by the product-limit method. The time interval for the above-mentioned end points was calculated from the first day of RT until the date of an event or of the last follow-up. The logrank test was used to assess the correlation of these end points with the expression of the cell-cycle-related factors, the clinical (age, T stage, and N stage), and therapeutic variables (addition of chemotherapy and OTT). Fisher's exact test (two-tailed) was used to evaluate differences in proportions. A difference with a *P*-value of less than 0.05 was considered significant.

## RESULTS

### Overall results

At the last follow-up, 50 patients were alive, 47 had died, and one was lost to follow-up. In all, 34 patients presented with one or more tumour-related events: 27 with persistent or recurrent local disease, 13 with regional disease (four alone), and seven with distant metastases (three alone). At 5 years, actuarial LC was 71% (95% CI: 62–80%), actuarial DFS was 64% (95% CI: 54–74%) and overall survival was 64% (95% CI: 54–73%).

### Immunostaining and clinical–pathological correlations

The distribution of the percentage of cells stained for the different factors studied is given in Table 2

**Table 2:** Distribution % of cells stained for the cell-cycle-related proteins

**Proteins**	**0%**	**0–5%**	**5–50%**	**50–90%**	**>90%**
Cyclin D1	65	24	8	1	0
Cyclin E	48	25	24	1	0
p21	7	27	46	18	0
p53	1	41	44	11	1
Mib1	0	8	50	36	4

**Figure 1:**
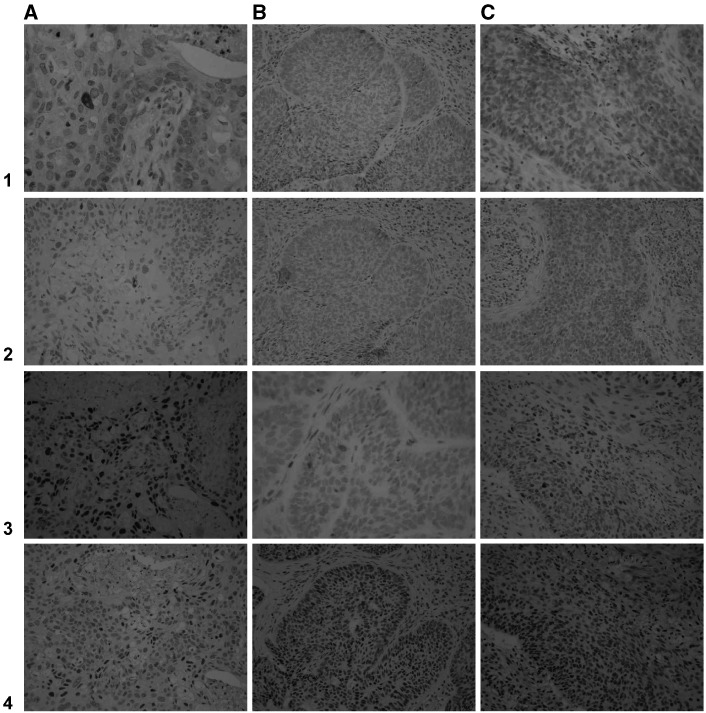
Results of immunohistochemical staining for 1=cyclin D1, 2=cyclin E, 3=p21, and 4=p53 in anal carcinomas. A large-cell carcinoma ((**A**)=6561/77), a small cell type ((**B**)=17386/78), and a carcinoma of mixed type ((**C**)=7917/80) are illustrated.

. High expression was found in 51% of tumours for cyclin E, in 34% for cyclin D1, in 65% for p21, in 57% for p53 (Figure 1

), and in 41% for Mib-1. Each factor was assessed for potential association with the following clinical–pathological parameters: age, sex, histological type, T category (T1–2/T3–4), N category (N0/N1–3), and tumour subsite. Except for an association of high Mib-1 with N1–3 category (*P*=0.004), no significant associations were found. On the other hand, assessment of the association between the different pathobiological factors showed a highly significant association of cyclin E with p21 expression (*P*<0.0001) and with p53 (*P*=0.014). A significant association of cyclin E with Mib-1 (*P*=0.008) and a trend towards an association with cyclin D1 expression (*P*=0.09) were also found. Moreover, a significant association was found between cyclin D1 and p53 (*P*<0.0001), while p21 expression was associated with Mib-1 expression (*P*=0.017). All the above associations were in the same direction and no significant inverse correlation was found.

### Univariate analysis

The results of the univariate analysis for LC and DFS using the expression of the different pathobiological factors studied, as well as the different clinical and therapeutic variables, are given in Table 3

**Table 3:** Univariate analysis of clinical, biological and therapeutic factors

	**No. of patients**	**% 5-year local control**	***P*-value**	**% 5-year DFS**	***P*-value**
*Age*					
⩽68/>68 years	48/50	68/74	0.44	55/72	0.08
					
*T category (UICC 1997)*					
T1–2/T3–4	60/38	81/55	0.006	73/49	0.01
					
*N category*					
N0/N1–3	74/24	80/44	0.0003	74/33	<0.0001
					
*Chemotherapy*					
No/yes	47/51	80/65	0.16	55/77	0.18
					
*Overall treatment time*					
⩽72/>72 days	50/48	84/58	0.008	77/50	0.004
					
*Immunostaining (CI)*					
Cyclin E low/high	48/50	74 (62–87)/69 (55–82)	0.54	67 (53–81)/61 (47–75)	0.56
Cyclin D1 low/high	65/33	73 (62–84)/68 (51–84)	0.56	67 (55–78)/57 (39–75)	0.40
p21 low/high	34/64	66 (49–82)/74 (63–85)	0.46	59 (41–76)/66 (54–78)	0.50
p53 low/high	42/56	78 (66–91)/66 (53–79)	0.22	75 (62–88)/55 (42–69)	0.07
Mib-1 low/high	58/40	75 (64–86)/66 (51–81)	0.37	67 (54–80)/59 (44–75)	0.30

DFS=disease-free survival, UICC=Union International Contre le Cancer; CI=confidence interval.

. Advanced T and N categories, as well as longer OTT, were the only variables that correlated significantly with low rate of LC and DFS, while a trend for a significant correlation was observed for high p53 expression and low DFS rate (*P*=0.07). Since the main aim of the study was to correlate the expression of cyclin E, cyclin D1, and p21 with LC and DFS, and no significant association with these factors was found in the univariate analysis, a multivariate analysis was not carried out.

### Subgroup analysis

In an attempt to define subgroups with wide differences in outcome, we grouped patients according to the most significant variables. The impact of each factor was studied after using stratification by the other biological factors or by splitting by clinical factors. By combining the different biological factors, no particular subgroup was identified to be associated with LC or DFS. According to the clinical subgroups, in the N0 category, patients with high p21 expression had a trend for a significantly higher 5-year LC (87%) compared to the group having low p21 expression (68%) (*P*=0.07). A similar result was observed when studying DFS, with the respective rates of 80 and 63% (*P*=0.1). No clear correlation was observed when assessing the other combinations.

## DISCUSSION

As in cervical cancer, there is strong evidence that HPV oncoproteins may be responsible for anal carcinogenesis (Frisch *et al*, 1997; Clarke and Chetty, 2001). The ability of viral oncoproteins to subvert cell-cycle checkpoints may constitute a mechanism by which viral oncoproteins induce genetic instability (Keating *et al*, 2001). HPV 16 E6 and E7 disrupt cell-cycle checkpoints, particularly affecting by different mechanisms nearly all CDK inhibitors linked to the G1 and G2 checkpoints (Galloway and McDougall, 1996). The discovery that increased levels of cyclin D1 and cyclin E promote cell growth by shortening the G1 phase (Resnitzky *et al*, 1994) led to the postulation that overexpression of those cyclins is positively involved in carcinogenesis and possibly in clinical tumour aggressiveness. On the other hand, evidence that p21 protein binds to and inhibits CDK–cyclin complexes, resulting in cell-cycle arrest during the G1/S transition, was consistent with the hypothesis that overexpression of this protein may be associated with less aggressive tumours (Harper *et al*, 1993).

We found that anal carcinomas express cyclin D1 in 34% (>5% of cells stained) and cyclin E in 51% of cases. Since this report is the first to evaluate those cyclins in this uncommon disease, no useful comparison with the results of other series is possible. Nevertheless, this expression is in line with the results reported in lung cancer (Anton *et al*, 2000) and cervical cancer (Keating *et al*, 2001). High expression of p21 was found in 64% of specimens, in accordance with the rate of 71% reported by Holm *et al* (2001) in a similar series of anal carcinomas. In contrast with the series reporting significant association between cyclin E expression and tumour stage (in breast cancer; Keyomarsi *et al*, 2002) or p21 expression and histology (in anal cancer; Holm *et al*, 2001), in the present series no association with the different clinical–pathological parameters was found. However, significant positive associations were observed between cyclin E and p21, p53 and Mib-1, as well as between cyclin D and p53. Although the positive association between cyclin E and Mib-1 (both related to proliferative activity) was not unexpected, the highly significant positive association between cyclin E and p21 was surprising, since their putative roles in cell-cycle regulation are presumably antagonistic. Further studies are required to determine whether these correlations reflect the complex role these factors may play in cell-cycle regulation or simply represent a spurious chance finding.

In the present study, we found no clear association between the expression of cyclin D1, cyclin E, and p21 with patient outcome as measured by LC and DFS. For completeness, the expression of p53 and Mib-1 was also included in our evaluation of cell-cycle-related factors, although these factors have been discussed in prior reports (Allal *et al*, 1997, 1998, 2002). Previous investigations of the prognostic value of cyclin E levels by using immunohistochemical techniques have produced conflicting results, for example, in breast cancer (Porter *et al*, 1997; Donnellan *et al*, 2001) and lung cancer (Anton *et al*, 2000; Muller-Tidow *et al*, 2001). However, the majority of studies observed a positive correlation between overexpression of cyclin E and poor outcome, including non-Hodgkin's lymphoma (Ferreri *et al*, 2001) and breast cancer (Keyomarsi *et al*, 2002). Whether this observation reflects publication bias to the detriment of negative studies remains speculative. Nevertheless, when interpreting the results of any retrospective study using immunostaining techniques, one should acknowledge their inherent limitations, namely the effect of long-term storage on molecular stability and the potential sampling bias related to variations in expression throughout the tumour specimen, which may alter the findings and consequently the conclusions.

Conflicting data were also reported concerning cyclin D1 expression, with overexpression associated with poor outcome in hypopharyngeal carcinomas (Nishimura *et al*, 1998), while for lung cancer cyclin D1 expression was reported to confer a better prognosis (Anton *et al*, 2000). These results are consistent with the as yet unclear role of cyclin D1 in cell proliferation processes. Indeed, it has been demonstrated that high levels of cyclin D1 occurring at the G1/S-phase junction arrest S-phase entry (Pagano *et al*, 1994), suggesting that the amount and timing of cyclin D1 can exert different effects on cellular proliferation. Thus, it seems that in different situations cyclin D1 can be either a positive or negative regulator of cellular proliferation.

The prognostic value of p21 expression has been reported by many studies, showing that underexpression of p21 protein is a negative prognostic marker in lung (Komiya *et al*, 1997), breast (Wakasugi *et al*, 1997), bladder (Stein *et al*, 1998), ovarian (Anttila *et al*, 1999), as well as in anal carcinomas (Holm *et al*, 2001). In our analysis of the entire series, no significant difference was observed in LC or DFS between subgroups of patients with tumours having high or low p21 expression. However, in the subgroup of 74 patients without clinical nodal involvement, a trend for a better LC and DFS was observed for tumours having high p21 expression. While these results may be considered to confirm the findings of Holm *et al* (2001), other studies have failed to reveal such a correlation (Zhang *et al*, 1995; Pellikainen *et al*, 2003), suggesting that the prognostic value of p21 still remains to be firmly established.

In conclusion, our findings indicate that pretreatment tumour expression of cyclin D1 and cyclin E has no apparent prognostic/predictive value in anal carcinomas. Moreover, no clear correlation was found between the expression of p21 protein and LC or DFS, even though in one subgroup (N0 patients) overexpression of p21 was associated with a tendency towards a more favourable outcome.
